# Supplementary material for: Trend of influenza before and during the COVID-19 pandemic in Nepal—A study from 2018 to 2022
Source: PLoS One. 2025 Apr 29;20(4):e0299610. doi: 10.1371/journal.pone.0299610 (PMC12040186; doi:10.1371/journal.pone.0299610)
Supplement: Attachment — Submitted filename: PONE-D-24-03169_reviewer (2) RY.pdf [file pone.0299610.s004.pdf]

PLOS ONE

Trend of Influenza before and during the COVID-19 pandemic in Nepal - a study from 2018 to 2022

--Manuscript Draft--

**Manuscript Number:**	PONE-D-24-03169
**Article Type:**	Research Article
**Full Title:**	Trend of Influenza before and during the COVID-19 pandemic in Nepal - a study from 2018 to 2022
**Short Title:**	Trend of Influenza before and during the COVID-19 pandemic in Nepal
**Corresponding Author:**	Priya N/A Jha, Master in Medical Laboratory Technology Microbiolo<br>World Health Organization<br>Lalitpur, NEPAL
**Keywords:**	Influenza, COVID-19, Public health, and social measures
**Abstract:**	A significant reduction in the incidence of Influenza during the early days of COVID-19 pandemic was reported worldwide. This study aims to understand the effect of COVID-19 pandemic response including the social and public health measures on the circulation of influenza in the country. This is the first national research of influenza seasonality patterns and epidemiology spanning five years, with a comparison between the pre-COVID-19 and COVID-19 periods. We have used the Influenza sentinel surveillance data in Nepal from 2018 to 2022 acquired from the National influenza Centre (NIC) at National Public Health Laboratory, Nepal. We also used the publicly available national COVID-19 case data released by the Ministry of Health and Population, Nepal. The data was analyzed for the trend of influenza cases, seasonality and the distribution of subtypes/lineages. Further, we compared the trend of Influenza with that of COVID-19 and the social and public health measures imposed in the country as part of the COVID-19 response. The average Influenza positivity rate has seen a drop from 39% to 14% during the COVID 19 period in comparison to Pre-COVID-19. Additionally, during the time of COVID 19 there has been a shift in the influenza bimodal seasonal pattern with one peak observed from July to October only. Influenza type A shows consistent dominance with variation of its subtype from year to year. Interestingly there was one reported case of Influenza A/H5 in 2019. This study shows that the influenza positivity rates decreased substantially after the COVID-19 pandemic began which may be related to the increasingly stringent public health and social measures implemented during the pandemic.
**Order of Authors:**	smriti shrestha, M.S.C MLT Microbiology and Immunology
	Priya Jha, Master in Medical Laboratory Technology Microbiolo
	Lilee Shrestha, Master
	Rashmi Mulmi, Master in Public Health
	Lok Bandhu Chaudhary, Master
	Runa Jha, MD Pathology
	ArunKumar Govindakarnavar, Phd
**Additional Information:**	
**Question**	**Response**
**Financial Disclosure**<br><br>Enter a financial disclosure statement that describes the sources of funding for the work included in this submission. Review the submission guidelines for detailed requirements. View published research	The author(s) received no specific funding for this work.

Powered by Editorial Manager® and ProduXion Manager® from Aries Systems Corporation

articles from *PLOS ONE* for specific examples.<br><br>This statement is required for submission and **will appear in the published article** if the submission is accepted. Please make sure it is accurate.<br><br>**Funded studies**<br>Enter a statement with the following details:<br>• Initials of the authors who received each award<br>• Grant numbers awarded to each author<br>• The full name of each funder<br>• URL of each funder website<br>• Did the sponsors or funders play any role in the study design, data collection and analysis, decision to publish, or preparation of the manuscript?<br><br>Did you receive funding for this work?	
**Competing Interests**<br><br>Use the instructions below to enter a competing interest statement for this submission. On behalf of all authors, disclose any competing interests that could be perceived to bias this work—acknowledging all financial support and any other relevant financial or non-financial competing interests.<br><br>This statement is required for submission and **will appear in the published article** if the submission is accepted. Please make sure it is accurate and that any funding sources listed in your Funding Information later in the submission form are also declared in your Financial Disclosure statement.<br><br>View published research articles from *PLOS ONE* for specific examples.	No

**NO authors have competing interests**<br><br>Enter: *The authors have declared that no competing interests exist.*<br><br>**Authors with competing interests**<br><br>Enter competing interest details beginning with this statement:<br><br>*I have read the journal's policy and the authors of this manuscript have the following competing interests: [insert competing interests here]*<br><br>* typeset	
**Ethics Statement**<br><br>Enter an ethics statement for this submission. This statement is required if the study involved:<br><br>• Human participants<br>• Human specimens or tissue<br>• Vertebrate animals or cephalopods<br>• Vertebrate embryos or tissues<br>• Field research<br><br>Write "N/A" if the submission does not require an ethics statement.<br><br>General guidance is provided below. Consult the submission guidelines for detailed instructions. **Make sure that all information entered here is included in the Methods section of the manuscript.**	Ethical approval was obtained from Nepal Health Research Council (Approval ID: 572-2022) to conduct the study.<br>This study is a retrospective study using National Public Health<br>data and Respimart data which is an open platform, and all data were fully anonymized and unidentified. Informed consent was<br>waived from both National Public Health Laboratory and Ethical ERB Nepal Health Research Council

Powered by Editorial Manager® and ProduXion Manager® from Aries Systems Corporation

**Format for specific study types**<br><br>**Human Subject Research (involving human participants and/or tissue)**<br>• Give the name of the institutional review board or ethics committee that approved the study<br>• Include the approval number and/or a statement indicating approval of this research<br>• Indicate the form of consent obtained (written/oral) or the reason that consent was not obtained (e.g. the data were analyzed anonymously)<br><br>**Animal Research (involving vertebrate animals, embryos or tissues)**<br>• Provide the name of the Institutional Animal Care and Use Committee (IACUC) or other relevant ethics board that reviewed the study protocol, and indicate whether they approved this research or granted a formal waiver of ethical approval<br>• Include an approval number if one was obtained<br>• If the study involved *non-human primates*, add *additional details* about animal welfare and steps taken to ameliorate suffering<br>• If anesthesia, euthanasia, or any kind of animal sacrifice is part of the study, include briefly which substances and/or methods were applied<br><br>**Field Research**<br><br>Include the following details if this study involves the collection of plant, animal, or other materials from a natural setting:<br>• Field permit number<br>• Name of the institution or relevant body that granted permission	
**Data Availability**<br><br>Authors are required to make all data underlying the findings described fully available, without restriction, and from the time of publication. PLOS allows rare exceptions to address legal and ethical concerns. See the PLOS Data Policy and FAQ for detailed information.	Yes - all data are fully available without restriction

Powered by Editorial Manager® and ProduXion Manager® from Aries Systems Corporation

A Data Availability Statement describing where the data can be found is required at submission. Your answers to this question constitute the Data Availability Statement and **will be published in the article**, if accepted.<br><br>**Important:** Stating 'data available on request from the author' is not sufficient. If your data are only available upon request, select 'No' for the first question and explain your exceptional situation in the text box.<br><br>Do the authors confirm that all data underlying the findings described in their manuscript are fully available without restriction?	
**Describe where the data may be found in full sentences. If you are copying our sample text, replace any instances of XXX with the appropriate details.**<br><br>• If the data are **held or will be held in a public repository**, include URLs, accession numbers or DOIs. If this information will only be available after acceptance, indicate this by ticking the box below. For example: *All XXX files are available from the XXX database (accession number(s) XXX, XXX.*).<br>• If the data are all contained **within the manuscript and/or Supporting Information files**, enter the following: *All relevant data are within the manuscript and its Supporting Information files.*<br>• If neither of these applies but you are able to provide **details of access elsewhere**, with or without limitations, please do so. For example:<br><br>*Data cannot be shared publicly because of [XXX]. Data are available from the XXX Institutional Data Access / Ethics Committee (contact via XXX) for researchers who meet the criteria for access to confidential data.*<br><br>*The data underlying the results presented in the study are available from (include the name of the third party*	All relevant data are provided within the manuscript.

and contact information or URL).<br>• This text is appropriate if the data are owned by a third party and authors do not have permission to share the data.<br><br>* typeset	
Additional data availability information:	

Manuscript

Click here to access/download;Manuscript;Final Trend of Influenza 2018 to 2022.docx

Trend of Influenza before and during the COVID-19 pandemic in Nepal - a study from 2018 to 2022

Smriti shrestha1*, Priya Jha2*, Lilee Shrestha1, Lok Bandhu Chaudhary1, Rashmi Mulmi 2, Arunkumar Govindakarnavar2, Runa Jha1

1 Department of National Influenza Center, National Public Health Laboratory, Kathmandu Nepal

2 World Health Emergency, World Health Organization, Kathmandu, Nepal.

Corresponding author email: Priya Jha (pjha@who.int)

These authors contributed equally to this work.

Abstract

A significant reduction in the incidence of Influenza during the early days of COVID-19 pandemic was reported worldwide. This study aims to understand the effect of COVID-19 pandemic response including the social and public health measures on the circulation of influenza in the country. This is the first national research of influenza seasonality patterns and epidemiology spanning five years, with a comparison between the pre-COVID-19 and COVID-19 periods. We have used the Influenza sentinel surveillance data in Nepal from 2018 to 2022 acquired from the National influenza Centre (NIC) at National Public Health Laboratory, Nepal. We also used the publicly available national COVID-19 case data released by the Ministry of Health and Population, Nepal. The data was analyzed for the trend of influenza cases, seasonality and the distribution of subtypes/lineages. Further, we compared the trend of Influenza with that of COVID-19 and the social and public health measures imposed in the country as part of the COVID-19 response. The average Influenza positivity rate has seen a drop from 39% to 14% during the COVID 19 period

in comparison to Pre-COVID-19. Additionally, during the time of COVID 19 there has been a shift in the influenza bimodal seasonal pattern with one peak observed from July to October only. Influenza type A shows consistent dominance with variation of its subtype from year to year. Interestingly there was one reported case of Influenza A/H5 in 2019. This study shows that the influenza positivity rates decreased substantially after the COVID-19 pandemic began which may be related to the increasingly stringent public health and social measures implemented during the pandemic.

Keyword: Influenza, COVID-19, Public health, and social measures

Introduction:

The influenza virus is well known for the pandemics it caused and its seasonal activity[1]. The World Health Organization (WHO) estimates that the influenza virus infection alone results -between 290,000 and 650,000 fatalities annually, with 36% of these deaths taking place in low- and middle-income nations[2]. Further, it is estimated that around 20% of the world's population is exposed to influenza virus each year, with 20-30% in pediatric and 5-10% among adult populations[3]. In Southeast Asia, the average annual death rate associated with influenza is very high, ranging from 3.5 to 9.2 per 100,000 people[4].

Coronavirus disease 2019 (COVID-19), caused by severe acute respiratory syndrome coronavirus 2 (SARS-CoV-2), was first reported in Wuhan, China, in December 2019[5]. The World Health Organization (WHO) declared the outbreak of COVID-19 public health emergency of international concern (PHEI) on January 30, 2020 and the pandemic on March 11, 2020[6]. In Nepal, the first case of COVID-19 was confirmed in January 2020, and the second case in March 2020, and sudden

increase was noted in May 2020[7]. The first wave of COVID-19 in Nepal peaked in late October 2020 [8] second wave due to delta variant in May 2021 and third wave peaked in Jan 2022[7]. The public health and social measures such as lockdown, and travel restrictions severely affected the mobility of people in Nepal in 2020 and the early half of 2021[10]. The public health and social measures put in place to control and mitigate COVID-19 pandemic largely affected Influenza surveillance globally including Nepal. Many countries that maintained influenza surveillance reported a decline in seasonal influenza activity during the early days of the COVID-19 pandemic[11]. While sentinel influenza surveillance (activities) in Nepal also significantly reduced during early days of COVID-19 pandemic, it was supplemented through testing SARS-CoV-2 PCR negative samples for Influenza following the WHO guidance[12].

The National Influenza Center (NIC) at the National Public Health Laboratory (NPHL) has been conducting Influenza surveillance at national level since 2010. This is a hospital-based sentinel surveillance including 15 hospitals selected based on geographical region and population density with the provision of sending representative samples on a weekly basis. Likewise, other collaborating laboratories share their aggregate Influenza test result to NIC to upload in Respimart platform. We analyzed the Influenza surveillance data of Nepal from 2018 to 2022 to understand the influenza trends before and during the COVID-19 pandemic.

Materials and Methods:

This descriptive cross-sectional study was done based on the influenza data of Nepal reported to Respimart platform. Respimart is data sharing platform in which the week wise aggregated Influenza test data is reported by the countries participating in Global Influenza Surveillance and Response system (GISRS). NIC, NPHL is responsible to report Influenza test data representing Nepal in Respimart. NIC maintains the database of the Influenza test done at NPHL and other

collaborating centers (sentinel and non- sentinel site) in MS-Excel to upload the data in Respimart weekly.

The aggregated data reported to Respimart from 2018 to 2022 were analyzed in MS-Excel to calculate the total number and percentage of samples processed, influenza positivity rate and types and subtypes (A/H1, A/H3, Victoria, Yamagata) on the basis of epi-week. Likewise, month wise distribution of the influenza cases was calculated to understand the Influenza seasonal pattern before and during COVID-19 period. The duration from 2018 to 2019 was considered as pre-COVID-19 period and from 2020 to 2022 was considered as during COVID-19 period. COVID-19 trend was plotted and compared with that of Influenza.

As the data submitted to Respimart includes only the aggregate data of Influenza test result received from the collaborating centers (or non-sentinel sites) which do not include the epidemiological data of the cases. Thus, we used the electronic data of specimens tested at NIC, NPHL from 2018 to 2022 to analyze the distribution of influenza in various age groups, gender and cases (SARI and ILI). The Chi-square tests were applied to test the association of influenza positivity in pre and during COVID-19 period.

Ethical approval was obtained from Nepal Health Research Council (Approval ID: 572-2022) to conduct the study. The data were accessed and analyzed in 2023 (March to May)

Results:

A total of 26,603 samples were reported in the Respimart from 2018 to 2022. However, age, and gender data, clinical history were available only from 18688 cases. (“Table 1 and 2”). The average positivity rate of influenza in Pre COVID is 39% whereas during COVID is 14%, indicating a

significant indicating a significant reduction (p value < 0.001) once the COVID-19 pandemic began in Nepal.

Table 1: Number of samples tested, positivity rate and subtype of Influenza circulation in Pre COVID and during COVID period.

												Positivity Rate (%)	P Value
			Influenza negative	Influenza Positive	Influenza Type A				Influenza Type B				
					H1	H3	H5	NOT SUBTYPED	VIC	YAM	NOT SUBTYPED		
	Year	Total Sample	N (%)	N (%)	N (%)	N (%)	N (%)	N (%)	N (%)	N (%)	N (%)		
PRE COVID	2018	3091	2134(69)	957 (31)	83(8.9)	491(52.7)	0	0	13(1.3)	175(18.8)	195(20.9)	39	<0.001
	2019	5783	3214(55.55)	2569(44.44)	1183(46)	498(19.3)	1(0.04)	18(0.7)	63(2.4)	24(0.9)	782(30.4)		
DURING COVID	2020	2915	2223(76.2)	692(23.7)	438(63.2)	223(32.2)	0	0	0	0	31(4.47)	14	
	2021	6777	5642 (83.2)	1135(16.7)	2(0.17)	608(53.5)	0	0	235(20.7)	0	290(25.5)		
	2022	8037	7332(91.2)	705(8.7)	362(51.3)	338(47.9)	0	0	1(0.14)	0	4(0.56)		

Table 2: Distribution of Influenza in different age group, gender, and clinical cases from NIC data (N=18688)

		Pre-COVID-19 (2018-2019)				During COVID-19 (2020-2022)			
		Negative	Positive	Total	% Positivity	Negative	Positive	Total	% positivity
**Age group**	<5	530	99	629	15.7	1117	129	1246	10.4
	5 to14	444	155	599	25.9	1610	155	1765	8.8
	15 to 65	2368	1248	3616	34.5	7873	677	8550	7.9
	≥65	836	284	1120	25.4	1095	68	1163	5.8
**Case**	ILI	1109	711	1820	39.1	6066	456	6522	7
	SARI	3069	1075	4144	25.9	5629	573	6202	9.2
**Gender**	Female	1942	865	2807	30.8	4750	449	5199	8.6
	Male	2236	921	3157	29.2	6945	580	7525	7.7

Influenza Type and subtype

The circulation of Influenza type A is found to be more prevalent in comparison to influenza type B throughout the given time period. During Pre-COVID-19 26% of influenza type A cases were detected, compared to 14% of type B cases, while during the COVID-19 period, there were 11.1% type A cases and 3.16% Type B cases. In year 2020 and 2022 the circulation of Influenza type B

has significantly dropped to less than 5% (shown in table 1), with Influenza type A predominating more than 95%. Among subtypes of Influenza A, variable pattern of dominance was seen year wise. Influenza A/H3 was the dominant strain in 2018 and 2021 whereas A/H1 was dominant in 2019-2020; and in year 2022 A/H1 and A/H3 were codominant strains. Remarkably, one Influenza A/H5 subtype was reported in the year 2019.

Month-wise pattern of influenza positivity of year 2018 to 2022:

There was a distinct pattern of peaks in two periods of the study, i.e., pre-covid and during covid time. In Pre-COVID year Influenza positivity was seen throughout the year with two peaks, one during January to March and another peak in July to October period. In contrast, during COVID-19 years, only single peak was observed from July to October as shown in "Fig 1 and 2"

Distribution of influenza in age groups, gender, ILI and SARI cases.

As described in Table 2 the influenza positivity rate was mainly in the adult age group 15-64 during Pre COVID year while it was higher in pediatric age group (0-14 years) during the COVID-19 period. Influenza positivity was significantly higher in ILI cases as compared to SARI cases in the year 2018, 2019, 2020 except in 2021. Gender wise, though male population was found to be more affected, comparatively no statistically significant differences were detected.

Fig 1: **Trend of Influenza during Pre-COVID (2018-2019) during COVID (2020-2022)**

Fig 2: Circulation of influenza subtype A and lineage B from 2018 to 2022

Fig 3: Month-wise pattern of influenza positivity of year 2018 to 2022

Fig 4: Influenza and COVID-19 Positivity of Nepal during 2018 to 2022.

Discussion:

Our study has shown that during Pre-COVID-19 years, influenza cases were seen throughout the year with dual peak, once in winter season (January to March) and in another rainy season (July to October). Other studies from Nepal from previous years have also shown year-round circulation of influenza virus with seasonal peaks[13,14].. The study by Wong NS et al from Hong Kong, China, also reported similar dual influenza peak: a winter peak that occur within initial 2 months of the year and a summer peak in the month of August or September [15].

In contrast, during the COVID-19 years 2020-2022 a significant decline in influenza cases was in Nepal seen ($p=<0.01$). Correspondingly, surveillance data globally have shown decline in influenza cases in response to the COVID-19 pandemic[16,17,18,19].

Overall, less number of influenza tests compared to previous year were performed in 2020 due to COVID-19 testing prioritization. However In the beginning of the year 2020, 35% of influenza cases were reported from epi week 1 to 13 and the number of tests gradually decreased as shown in fig 1. One of the main reasons behind this is probably the announcement of nationwide lockdown from March 24, 2020 by the Government of Nepal with cessation of domestic travel, physical distancing, and use of personal protective measures[20]. However, surge in influenza testing was witnessed in 2021-2022 with re-emphasis on influenza, though the positivity rate was still substantially lower (2021: 16.7% & 2022: 8.7%). A noteworthy observation is that the upsurge of COVID-19 wave in Nepal coincides with decreased influenza positivity during years 2020-2022. Study by Young Lu et. al also stated similar finding in China that displayed a significant decline, in influenza cases coinciding with the COVID-19 pandemic[21]. Similar trend was seen in various other countries like Japan, Thailand, USA, and Xinxiang China where seasonal influenza circulation was lower in 2020 than in preceding years[22,23,24,25]. Canada also reported overall lower

percentage of tests positive for all seasonal respiratory viruses including influenza A and B during 2020-2021 compared to pre-pandemic seasons[26].

In addition to decreased positivity rate, COVID-19 has affected the regular seasonal cycle of influenza circulation in Nepal as well. During Covid 19 period only a single peak of influenza was seen from July to October in contrast to regular biannual peak seen before COVID-19 pandemic. Similar findings with late and unusually protracted influenza seasons have been reported in the Northern Hemisphere[27]. This has further added to the finding that influenza seasonality has varied in comparison to pre covid years.

Widespread use of public health interventions to prevent COVID -19 pandemic may be one of the factors explaining rapid declines in influenza cases as both the diseases have common route of transmission which may significantly lower the risk of influenza exposure[18]. Furthermore, respiratory samples were prioritized for COVID□19 testing and only those tested negative were sent for influenza testing. Few dual positive cases may have also been missed using this approach. Similar studies in the USA have shown that implementation of public health measures such as social distancing, travel restrictions, use of face masks, and frequent hand washing has contributed to such observations[28]. Study by Yiman Geng et al showed that there was significant reduction of the spread of the influenza virus following public health interventions for COVID-19 in China)[29].

One of the limitations of our study is that we were able to present limited epidemiological data, i.e, the age- and gender-specific data that was available in the NIC, NPHL databases only. Epidemiological data such as age and gender, distribution are not available in Flumart so complete data regarding the same was not available. However, available data has shown that there is a significant difference in the age -wise positive cases pre-covid and post-covid. Our results demonstrate higher positivity in the age groups of 15-64 during Pre-COVID -19 and 0-14 during

COVID-19. Also, in terms of Influenza B lineage determination, we found Yamagata to be the predominate lineage in 2018, dropping dramatically in the next year, and consistently no cases reported from the year 2020 to 2022. Study by Zoltan et al and Marios et.al[30] reported no new isolate of Yamagata lineage of influenza B virus since March 2020 and probably no longer exist. However, this analysis revealed that the majority of Influenza B was not routinely subtyped to prove that Yamagata was not still in circulation.

Conclusion: The COVID-19 pandemic and the public health and social measured imposed in response to it had a significant impact on the circulation of the Influenza virus in Nepal. As soon as the lockdown and other PHSM were enforced there was a drastic decline in Influenza circulation significantly changing the seasonality of Influenza. This shows that the PHSM measures used in response to COVID-19 were significantly effective against controlling transmission of Influenza. The droplet mode of transmission of Influenza would have been the reason for this finding. While Influenza surveillance was significantly affected during COVID-19 pandemic in many countries, Nepal adopted the WHO recommendations and used COVID-19 PCR negative samples and retrospectively tested for Influenza to bridge the surveillance gap. Hence the Influenza surveillance data is reliable and add strength to the findings of this study. Overall, it is clear that the PHSM used during COVID-19 pandemic was very effective in controlling Influenza transmission and we could implement them to control the influenza viral transmission during seasonal influenza epidemics . Also, countries should adapt systems to ensure continued Influenza surveillance is warranted even in global public health emergencies.

Disclaimer

This work represents the personal opinion of the authors and not that of the World Health Organization and the Ministry of Health and Population, Government of Nepal.

Acknowledgment

We would like to express our sincere gratitude to WHO Country office Nepal for organizing manuscript writing workshop.

Authors' Contribution

Conceptualization: PJ, RJ, SS

Methodology: PJ, RM, SS

Data collection/ cleaning: PJ, RM, LBC

Data analysis: AG, PJ, RM, SS, LBC

Data curation: PJ, RM, LBC

writing—original draft preparation: PJ, SS

writing—review and editing: AG, LS, RJ

supervision: AG , RJ

(RJ: Runa Jha, AG : Arun Kumar Govindakarnavar, SS : Smriti Shrestha, PJ: Priya Jha, LS: Lilee Shrestha, RM; Rashmi Mulmi, LCB: Lok Bandhu Chaudhary)

All authors have read and agreed to the published version of the manuscript.

Funding

We did not receive any funding for this research /data analysis.

References:

1. Types of Influenza Viruses, Center for Disease control and Prevention. https://www.cdc.gov/flu/about/viruses/types

2. Salman M, Badar N, Ikram A, Nisar N, Farooq U. Estimation of seasonal influenza disease burden using sentinel site data in Pakistan 2017–2019: A cross□sectional study. Influenza and Other Respiratory Viruses. 2023 Mar;17(3):e13125.

3. Fischer WA, Gong M, Bhagwanjee S, Sevransky J. Global burden of influenza: contributions from resource limited and low-income settings. Global heart. 2014 Sep;9(3):325.

4. Malik YA. IMPACT OF INFLUENZA IN SOUTH-EAST ASIA. International Journal of Infectious Diseases. 2023 May 1;130: S40-1.

5. Mohanty SK, Satapathy A, Naidu MM, Mukhopadhyay S, Sharma S, Barton LM, Stroberg E, Duval EJ, Pradhan D, Tzankov A, Parwani AV. Severe acute respiratory syndrome coronavirus-2 (SARS-CoV-2) and coronavirus disease 19 (COVID-19)–anatomic pathology perspective on current knowledge. Diagnostic pathology. 2020 Dec;15:1-7.

6. World Health Organization. COVID 19 Public Health Emergency of International Concern (PHEIC). Global research and innovation forum: towards a research roadmap.

7. Pandey BD, Ngwe Tun MM, Pandey K, Dumre SP, Nwe KM, Shah Y, Culleton R, Takamatsu Y, Costello A, Morita K. How an outbreak of COVID-19 circulated widely in Nepal: a chronological analysis of the national response to an unprecedented pandemic. Life. 2022 Jul 20;12(7):1087.

8. Traversing Nepal's COVID Peaks and Valleys https://www.thinkglobalhealth.org/article/traversing-nepals-covid-peaks-and-valleys by by Meghnath Dhimal, Krishna Aryal, Achyut Raj Pandey, June 9, 2021

9. Lessons from the second wave: Reference for averting the third wave, The Himlayan Time June 2021 https://thehimalayantimes.com/opinion/lessons-from-the-second-wave-reference-for-averting-the-third-wave

10. Poudel A, Lamichhane A, Gautam S, Ghimire GD, Sharma S. Recurrent COVID-19 Waves and Lockdown: Impact on Daily Life and Mental Health of People in Nepal. Mental Illness. 2023 Jan 25;2023.

11. Parums DV. A decline in influenza during the COVID-19 pandemic and the emergence of potential epidemic and pandemic influenza viruses. Medical Science Monitor: International Medical Journal of Experimental and Clinical Research. 2021;27:e934949-1.

12. Integration and expansion: Leveraging influenza systems for the COVID-19 response 3 February 2022 Departmental news Reading. https://www.who.int/news/item/03-02-2022-integration-and-expansion-leveraging-influenza-systems-for-the-covid-19-response

13. Jha BK, Pandit R, Jha R, Manandhar KD. Overview of seasonal influenza and recommended vaccine during the 2016/2017 season in Nepal. Heliyon. 2020 Jan 1;6(1).

14. Upadhyay BP, Ghimire P, Tashiro M, Banjara MR. Characterization of Seasonal Influenza Virus Type and Subtypes Isolated from Influenza Like Illness Cases of 2012. Kathmandu Univ Med J. 2017;57(1):56-60.

15. Wong NS, Leung CC, Lee SS. Abrupt subsidence of seasonal influenza after COVID-19 outbreak, Hong Kong, China. Emerging Infectious Diseases. 2020 Nov;26(11):2752.

16. Akhtar Z, Chowdhury F, Rahman M, Ghosh PK, Ahmmed MK, Islam MA, Mott JA, Davis W. Seasonal influenza during the COVID-19 pandemic in Bangladesh. PLoS One. 2021 Aug 3;16(8):e0255646.

17. Dhanasekaran V, Sullivan S, Edwards KM, Xie R, Khvorov A, Valkenburg SA, Cowling BJ, Barr IG. Human seasonal influenza under COVID-19 and the potential consequences of influenza lineage elimination. Nature communications. 2022 Mar 31;13(1):1721.

18. Soo RJ, Chiew CJ, Ma S, Pung R, Lee V. Decreased influenza incidence under COVID-19 control measures, Singapore. Emerging infectious diseases. 2020 Aug;26(8):1933.

19. Olsen SJ, Azziz-Baumgartner E, Budd AP, Brammer L, Sullivan S, Pineda RF, et al. Decreased Influenza Activity During the COVID-19 Pandemic — United States, Australia, Chile, and South Africa, 2020. 2020;69(37).

20. Sharma K, Banstola A, Parajuli RR. Assessment of COVID-19 pandemic in Nepal: A lockdown scenario analysis. Frontiers in public health. 2021 Apr 8;9:599280.

21. Lu Y, Wang Y, Shen C, Luo J, Yu W. Decreased incidence of influenza during the COVID-19 pandemic. International Journal of General Medicine. 2022 Mar 14:2957-62.

22. Sakamoto H, Ishikane M, Ueda P. Seasonal influenza activity during the SARS-CoV-2 outbreak in Japan. Jama. 2020 May 19;323(19):1969-71.

23. Suntronwong N, Thongpan I, Chuchaona W, Budi Lestari F, Vichaiwattana P, Yorsaeng R, Pasittungkul S, Kitphati R, Vongpunsawad S, Poovorawan Y. Impact of COVID-19 public health interventions on influenza incidence in Thailand. Pathogens and global health. 2020 Jul 3;114(5):225-7.

24. Olsen SJ, Winn AK, Budd AP, Prill MM, Steel J, Midgley CM, Kniss K, Burns E, Rowe T, Foust A, Jasso G. Changes in influenza and other respiratory virus activity during the COVID-19 pandemic—United States, 2020–2021. Morbidity and Mortality Weekly Report. 2021 Jul 7;70(29):1013.

25. Lin Zeng 1, Feng Zang 2, Ninghong Song 3 4, Zhanjie Li 2 Analysis of influenza trend and impact of COVID-19 in Kezhou, Xinjiang for 8 consecutive years

26. Groves HE, Piché-Renaud PP, Peci A, Farrar DS, Buckrell S, Bancej C, Sevenhuysen C, Campigotto A, Gubbay JB, Morris SK. The impact of the COVID-19 pandemic on influenza, respiratory syncytial virus, and other seasonal respiratory virus circulation in Canada: A population-based study. The Lancet Regional Health–Americas. 2021 Sep 1;1

27. Lee SS, Viboud C, Petersen E. Understanding the rebound of influenza in the post COVID-19 pandemic period holds important clues for epidemiology and control. International Journal of Infectious Diseases. 2022 Sep 1;122:1002-4.

28. Zipfel CM, Bansal S. Assessing the interactions between COVID-19 and influenza in the United States. medRxiv. 2020 Apr 1:2020-03.

29. Geng Y, Li G, Zhang L. The impact of COVID-19 interventions on influenza and mycobacterium tuberculosis infection. Frontiers in Public Health. 2021 May 21;9:672568.

30. Vajo Z, Torzsa P. Extinction of the Influenza B Yamagata Line during the COVID Pandemic—Implications for Vaccine Composition. Viruses. 2022 Aug 9;14(8):1745.

Figure 1

Click here to access/download;Figure;fig 1.tif

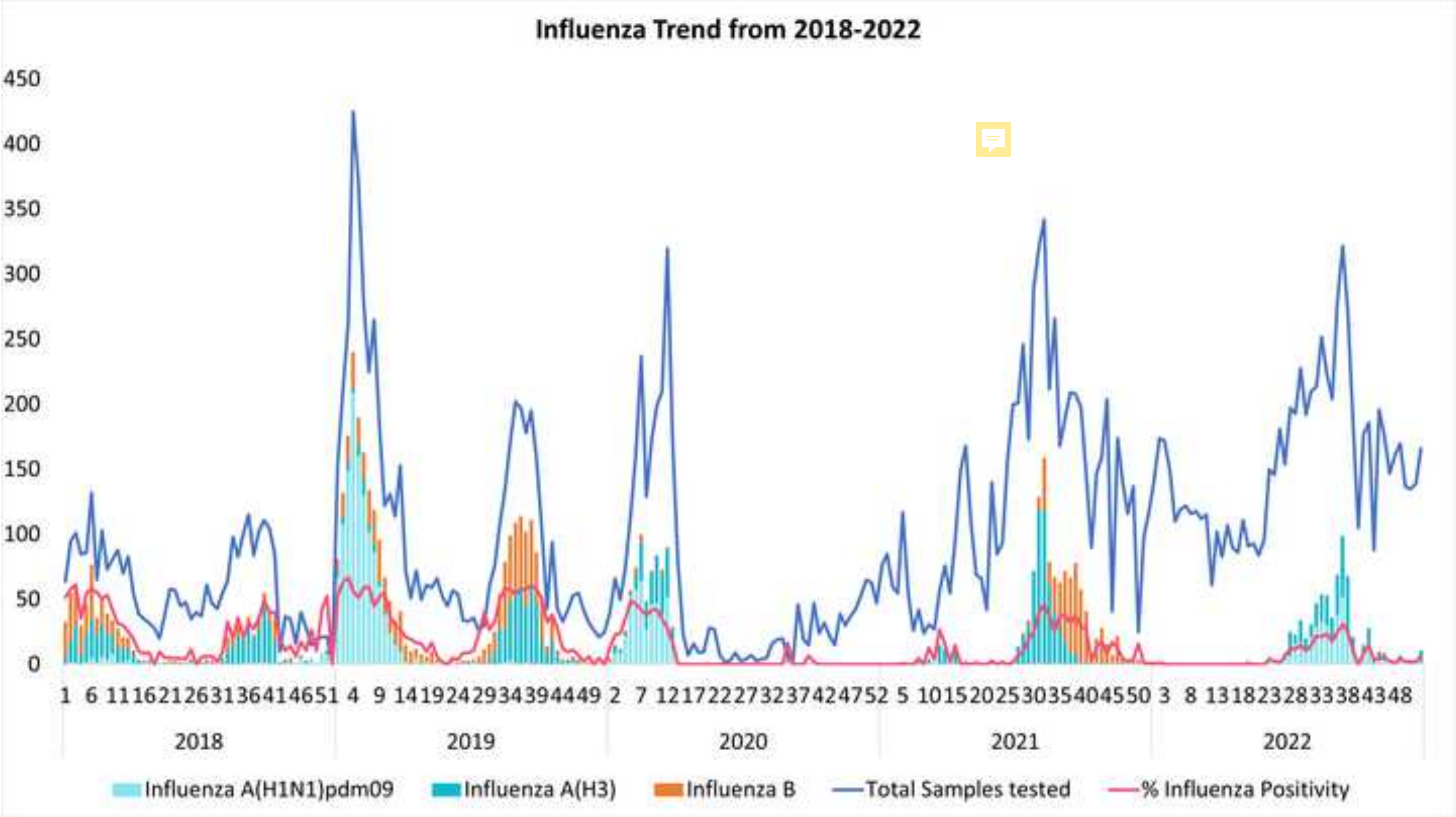

Figure 2

Click here to access/download;Figure;Fig 2 .tif

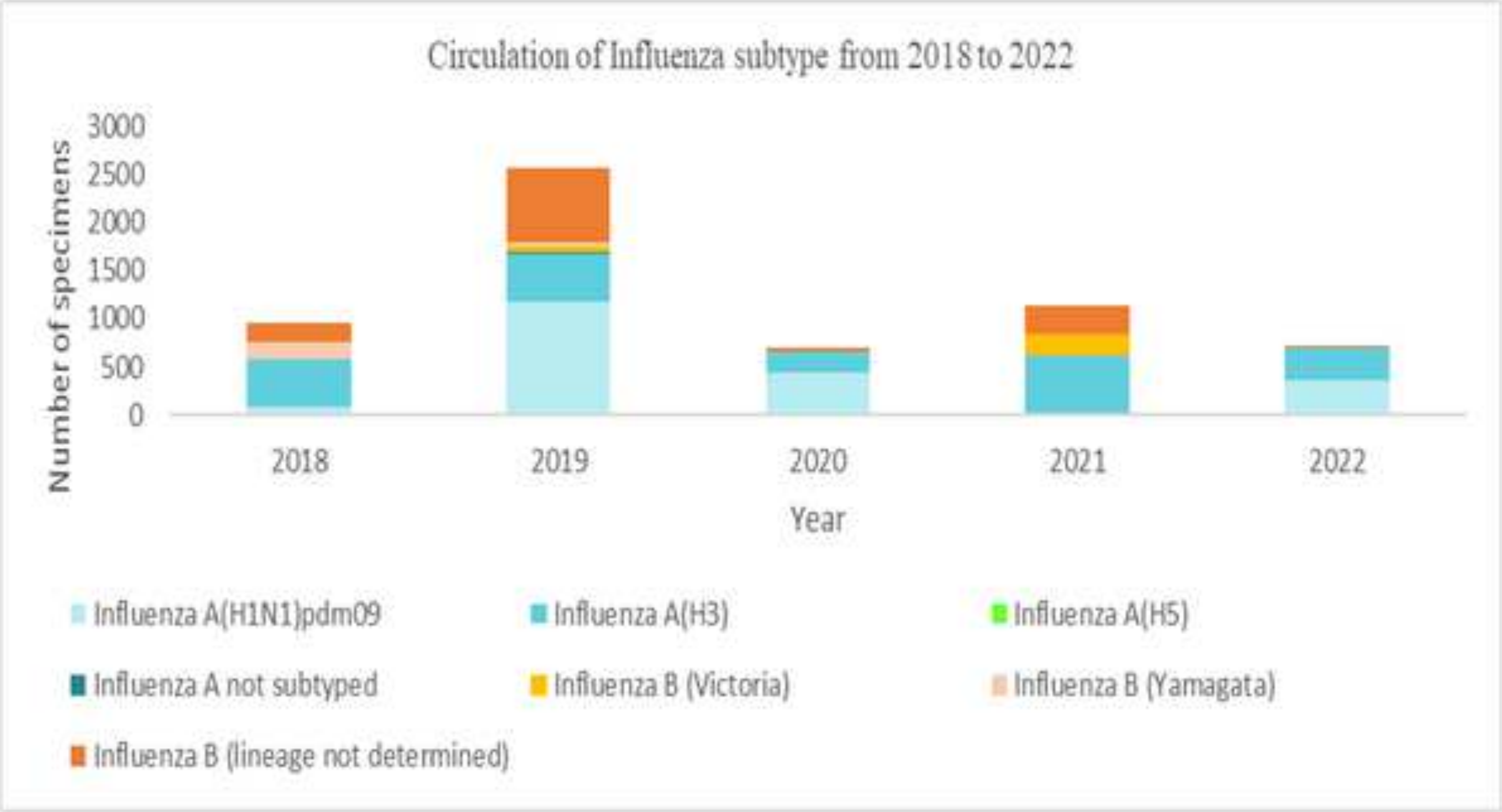

Figure 3

Click here to access/download;Figure;Fig 3 .tif

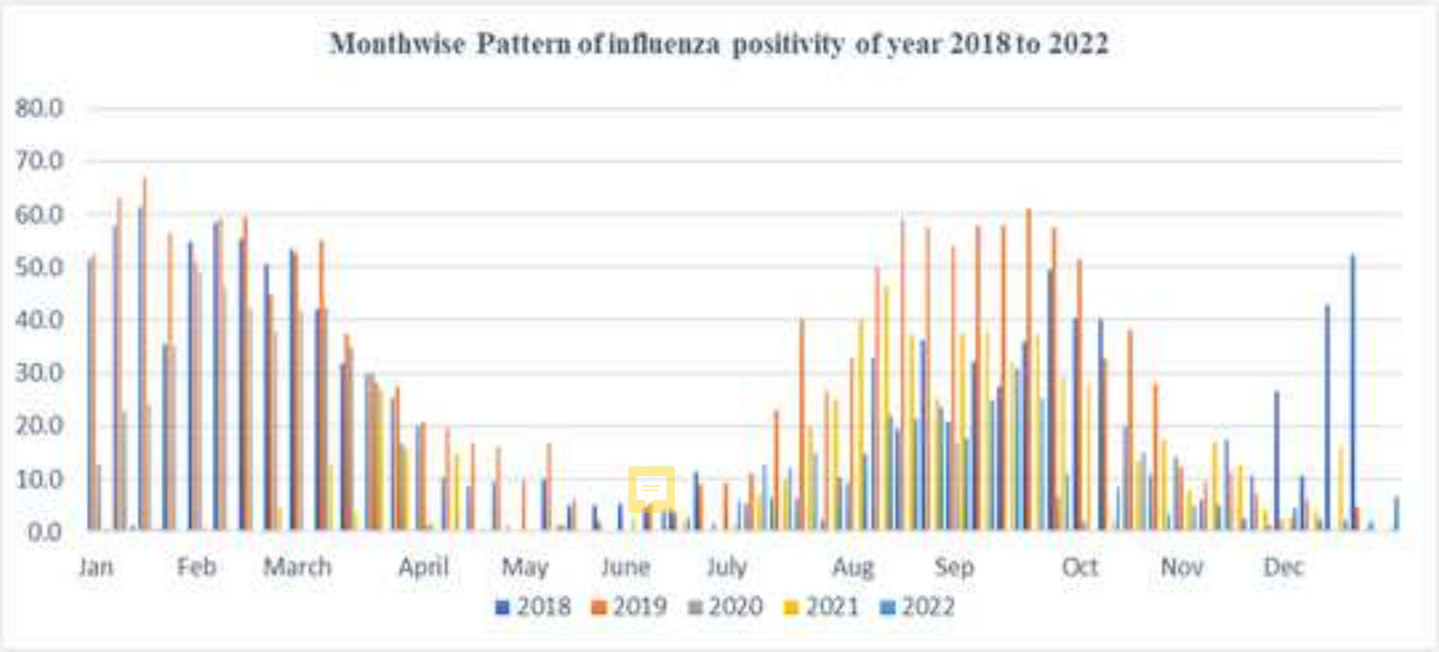

Figure 4

Click here to access/download;Figure;fig 4 influenza tgif .tif

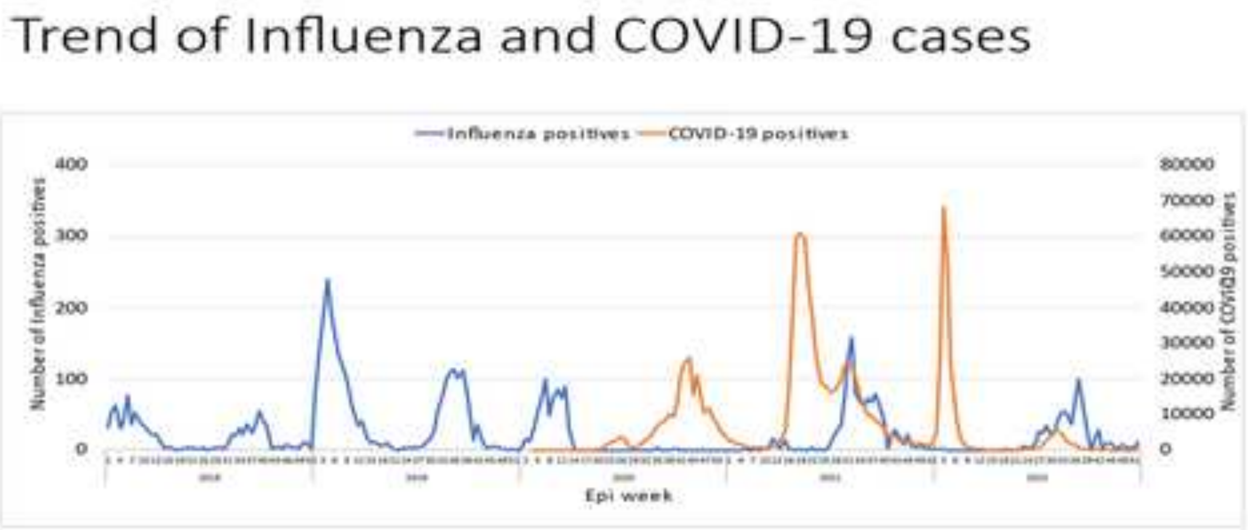

Gantt Chart

Click here to access/download
Supporting Information
gantt chart influenza .docx
