# Supplementary material for: Trend of influenza before and during the COVID-19 pandemic in Nepal—A study from 2018 to 2022
Source: PLoS One. 2025 Apr 29;20(4):e0299610. doi: 10.1371/journal.pone.0299610 (PMC12040186; doi:10.1371/journal.pone.0299610)
Supplement: Attachment — Submitted filename: Final Rebuttal Letter response to reviwers to submit .docx [file pone.0299610.s007.docx]

Dear Editor,

Plos One,

Subject: Response to the reviewers and academic reviewers comment

Thank you for the opportunity to revise our manuscript titled “Trend of Influenza Before and During the COVID-19 Pandemic in Nepal: A Study from 2018 to 2022” (PONE-D-24-03169). We appreciate the time and effort that you and the reviewers have invested in evaluating our work. We have carefully considered all the comments and suggestions provided and have made substantial revisions to address each point raised.

Please find the comments and response from the reviewer as follows:

**Reviewers Comment and response**

Reviewer #4

1. Comment : Abstract:

The objective could be redefined as: “This study aims to understand the impact of public health and social measures implemented during the COVID-19 pandemic on influenza circulation in Nepal.”

- Response: Added, line no: 12-13

2. Comment: Please provide data to support the following statement: “Additionally, during the time of COVID-19 there has been a shift in the influenza bimodal seasonal pattern, with only one peak observed. Influenza type A consistently dominated, with variations in its subtype observed from year to year.”

- Response: The data supporting this is provided in the Fig 1A and Fig 1B

3. Comment: While the conclusion statement is clear, it could be more impactful by linking it to broader implications for future public health measures. For example, focus on precise and actionable recommendations, such as how these findings inform public health policy or enhance pandemic preparedness.

- Response: Added, line no: 27-30

4. Comment: Manuscript:

Introduction of the manuscript: The objective statement could be refined as:“This study aims to understand the impact of public health and social measures implemented during the COVID-19 pandemic on influenza circulation in Nepal from 2018 to 2022.”

- Response: Added, line no 71-73

5. Comment: The following statement should be moved to the Methods section:“The period from 2018 to 2019 was considered the pre-COVID-19 period, while from 2020 to 2022 was considered the COVID-19 period.”

- Response: Added, line no 76-77

6. Comment: Method:

Present case definitions for ILI and SARI in a separate paragraph under the subheading “Case Definition.”

- Response: separate paragraph for case definition given. Line no: 92-97

7. Comment: In line 98, replace “A (H3N2/H1N1pdm09)” with “Influenza A(H1N1)pdm09 and Influenza A(H3N2)” for clarity.

- Response: It is PCR reagent name provided by CDC for molecular detection of influenza Hence, the name is kept as it is (CDC Influenza Virus Real-Time RT-PCR panel, Influenza A (H3N2/H1N1pdm09) subtyping panel).

8. Comment: Correct the sentence starting with “Subtyping of positive influenza B specimens...” for accuracy.

- Response: necessary changes made. Line no: 107

9. Comment: Mention the specific statistical tests used for analyzing trends and comment on their appropriateness for the dataset.

- Response: The specific statistical tests used for analyzing trends was Chi-square test to test the association between two categorical variables (i.e influenza positive, negative and pre and during COVID-19 trends mentioned in line number 81 to 88.

10. Comment: In lines 73–76, consider rewriting or splitting the sentence for better clarity, as it is currently hard to understand.

- Response: Necessary changes made. Line no: 81-86

11. Comment: Revise for grammatical consistency and clarity. For example, in lines 105–106,revise:“The average positivity rate of influenza in pre-COVID-19 is 39% whereas during COVID-19 is 14%.” to “The average influenza positivity rate was 39% pre-COVID-19, compared to 14% during COVID-19.”

- Response: Necessary revision done and changes made. Line no: 115-116

12. Comment: Avoid starting sentences with abbreviations. For example, in line 176, instead of: “PHSM were enforced there was a drastic decline in Influenza circulation...” revise to: “After public health and social measures (PHSM) were enforced, there was a drastic decline in influenza circulation...”

- Response: revise to: Necessary changes made. Line no: 211-212

13. Comment: Avoid writing “influenza” with a capital letter unless it is the first word of a sentence.

- Response: Changes made where applicable.

14. Comment: Ensure consistent use of proper punctuation and capitalization throughout the manuscript (e.g., “During COVID-19” instead of “DURING COVID-19”).

- Response: Necessary changes made.

Reviewer #5:

1. Comment: The author’s conclusion largely relies on surveillance data, which was omitted from the text. Therefore, I would recommend that the author provide more information about the surveillance system, including the number of sites, locations, etc in the Methods section.

- Response: More information provided on surveillance system in method section. Line no: 78-81,

Introduction section. Line no: 57-61

Discussion section: 185-191

2. Comment: The changes in the performance of the surveillance system may affect the author’s conclusion. Therefore, I would strongly recommend the performance changes of surveillance during the COVID-19 pandemic as a limitation of the study, referencing two literatures (PMID: 39173559 and PMID: 39045828).

- Response: Addressed as the limitation of the study in the discussion section in line number 219 to 226

3. Comment: Please elaborate on other studies conducted in Asian countries in the discussion.

- Response: Studies conducted in east and southeast Asia, Malaysia reported in line no: 199-202 and line no: 204-206.

We have completed the revisions and made the necessary adjustments to address the reviewers' and academic concerns, aiming to improve the manuscript. We trust the revised version meets with your approval. Please let us know if any further clarification is needed. Thank you once again for your consideration.

Thank you,

Regards

Priya Jha.
